# A commentary on ‘Addressing Africa’s outrageous neurosurgeons deficit: what could the problem be?’

**DOI:** 10.1097/JS9.0000000000000703

**Published:** 2023-10-11

**Authors:** Si-Un Frank Chiu, Kang Lu, Chong-Chi Chiu

**Affiliations:** a Department of Computer Science; b Department of Economics, Brown University, Providence, Rhode Island, USA; c School of Medicine, College of Medicine; d Department of Neurosurgery, E-Da Hospital; e Department of Neurosurgery; f Department of General Surgery; g Department of Medical Education and Research, E-Da Cancer Hospital, I-Shou University, Kaohsiung, Taiwan

*Dear Editor*,

Wireko *et al*.^1^ examined the state of the neurosurgical workforce in Africa, highlighting the significant workforce deficits and training challenges, particularly in regions like Central, East, and West Africa. Despite increasing training programs, there still needs to be more neurosurgeons, around 9000 to meet the demand^2^. Besides, there are many challenges in education provision in Africa. This serious insufficiency of neurosurgical care and education has raised public awareness, which requires detailed investigation and urgent action.

Postgraduate education in neurosurgery in Africa has two imperative main goals: improving current medical service delivery and training future neurosurgeons. Many African medical students are interested in neurosurgery. However, apart from the immature mentorship structures and scarcity of correct and detailed information^1^, unequal access, lack of facilities, and insufficient training programs and resources, unfair pay and benefits all hinder aspiring neurosurgeons’ career choices.

Dada *et al*.’s study investigates the status and differences in neurosurgical residency programs across Africa. Their research found notable variations in the curricula, recruitment processes, evaluation methods, and content of these programs^3^.

Besides, African neurosurgery, particularly in sub-Saharan Africa, still lacks adequate representation of women^2^. Esene *et al*. surveyed African neurosurgery residents, fellows, and consultants to understand their challenges and needs. It found that while essential neuroimaging resources were relatively accessible, there were limitations in access to neurorehabilitation and operative equipment, limiting hands-on training opportunities^4^. Inadequate training program length, curriculum, available resources, variation in the syllabus, and lack of perfect mentorship and standard assessment severely impact the quality of training and subsequent service delivery. Many patients need access to nearby neurosurgical centers, exacerbating the issue of limited resources and out-of-pocket expenses for patients.

Initiatives from organizations like the World Federation of Neurosurgical Societies (WFNS) and the Continental Association of African Neurosurgical Societies (CAANS) strive to improve access to neurosurgical care in Africa. In addition to the main training system for neurosurgeons in African countries, residents and fellows could receive better specialty training outside Africa, often in European neurosurgery departments. Although this provides an essential training pillar for the development of neurosurgery in Africa, some trainees might face the problems of inadequate duration and subpar training provided by certain departments. Besides, inadequately prepared candidates would result in the emergence of neurosurgeons with minimal proficiency, unable to make meaningful contributions to the advancement of neurosurgery upon return to their home countries. Moreover, there is also a challenge of these neurosurgery trainees readjusting when they go back to work in their native countries. Often, the conditions in their home countries significantly differ from those they became accustomed to during their training period. This disparity might lead to disillusionment and a notable number of well-trained neurosurgeons opting to leave the field of neurosurgery in their respective nations, and even boosting their incentive of emigration.

Collaborations between African training centers and non-governmental or academic organizations, often from high-income countries, are helping to bridge gaps in neurosurgical education and practice. Esene *et al*.’s study emphasized the need for investments in equipment and neurorehabilitation to support training and professional development^4^. Besides, targeted efforts should be on creating a pathway for women who have an interest in pursuing neurosurgery within these regions.

The significant obstacles confronting rural healthcare in Africa mainly involve substantial deficits in healthcare infrastructure. Consequently, there is a pressing need for considerable investments in healthcare infrastructure within these areas, which includes initiatives like building additional rural hospitals with governmental or external backing. While improving neurosurgery availability demands significant investment, it’s also essential to formulate strategies for delivering cost-effective neurosurgical education and care at regional and rural levels^5^.

With the assistance of the internet, telecommunication, and advanced artificial intelligence technology, they provide more cost-effective solutions like online education resources, participation of research seminar courses, workshops, digital education modules, operative videos, and tele-simulation to improve training and research capabilities. Collaboration among low- and middle-income countries and high-income institutions is encouraged, and partnerships should be monitored for effectiveness. Despite the challenges, there is evidence of positive evolution in neurosurgical practice in Africa, supported by the efforts of organizations like WFNS and CAANS and their respective youth committees^4^.

Neurosurgery training in Africa began later than in other continents and has been regionalized, especially in South and North African countries. Although African neurosurgery has made progress, concerted efforts from governments, neurosurgeons, societies, training programs, and international associations are needed to bridge gaps between the actual neurosurgical capacity and unmet needs. Besides, advocacy structures are recommended to influence policy and resource allocation for neurological health at national and continental levels.

## Ethical approval

This is only a commentary, not research involving patients. No ethical approval is required.

## Consent

This is only a commentary, not research involving patients. No patient consent is required.

## Sources of funding

This is a commentary. We have no funding for our commentary.

## Author contribution

S.-U.F.C.: conceptualization and writing the original draft; K.L.: validation; C.-C.C.: conceptualization, supervision, submission, and correspondence.

## Conflicts of interest disclosure

There are no conflicts of interest in our commentary.

## Research registration unique identifying number (UIN)

This is a commentary to a study published in ‘International Journal of Surgery’. No UIN is required.

## Guarantor

Professor Chong-Chi Chiu.

## Data availability statement

This is only a commentary to a study published in ‘International Journal of Surgery’. There is no research data in our commentary.

## Provenance and peer review

This paper was not invited.
